# The Final Court of Appeal

**Published:** 1916-09-30

**Authors:** 


### THE FINAL COURT OF APPEAL.

The whole system of compulsory military service in this country is of so recent an adoption that imperfections in its workings are natural enough, even if some of them could have been averted by reasonable foresight on the part of the executive. During the last two or three months a part of the whole scheme which has come in for an exceptional amount of public criticism is the proceedings of the Medical Boards set up as part of the administration of the Act. That much of this criticism has been undeserved is well known to the medical profession; although some Boards may have made more errors than others, the average of efficiency has been high, and probably the Boards have made fewer blunders than the lay Tribunals which have reviewed their decisions. However, since mistakes there admittedly have been, it is just as well to establish a final Court of Appeal to review professionally the verdicts of the Boards. In spite of the drawback of burdening an already over-cumbersome scheme with one more statutory authority, the idea of the Central Medical Board now established is sound.

The constitution of the Board, too, has been framed on the right lines. It consists of a senior officer of the Army Medical Service, of an expert consulting surgeon, and of an expert consulting physician. The first of the trio, Colonel E. H. L. Lynden-Bell, will no doubt bring to the deliberations of the Board that expert knowledge of the relative strains exacted by different categories of Army service, which only a highly experienced professional Army medical officer can supply. The surgeon, Sir Frederick Treves, has so high a reputation that his decisions on surgical matters are hardly likely to be questioned even by the yellowest of the daily press: though he has so much other work to do that this extra duty is one he might, from some points of view, have been excused. The physician, Sir James Mackenzie, is equally eminent, and will have the lion's share of the work, it may be anticipated. A very large number of the cases that have led to disagreements between Medical Boards and local Tribunals have been alleged cases of heart disease; and we know of no one more fitted to cope with these than Sir James Mackenzie. It is too often overlooked by the glib critics in the halfpenny press that large numbers of reluctant recruits—more shame to them—have deliberately "doped" themselves with drugs calculated to upset temporarily the equilibrium of their hearts. When these wiles have failed to deceive military Medical Boards, these gentry are very fond of going to unsuspicious civilian doctors and of obtaining from them strongly worded certificates of unfitness. These certificates have been accepted at face value by local Tribunals, which are apt to be impressed by the influential names of some of those who have signed them in many cases. There is reason to believe, unfortunately, that septuagenarian consultants, whose names carry more weight with the public than with the profession, have been much too guileless and gullible in this respect; largely, no doubt, through unfamiliarity with the researches on heart diseases of Sir James Mackenzie and his school of followers within the past ten or fifteen years.

